# Pelvic Radiation-Induced Sarcoma With Rhabdomyoblastic Differentiation Following Treatment of Cervical Cancer

**DOI:** 10.7759/cureus.15428

**Published:** 2021-06-03

**Authors:** Shahd S Almohsen, Hala Alnuaim, Alaa A Salim, Haitham Arabi

**Affiliations:** 1 Department of Pathology and Laboratory Medicine, King Abdulaziz Medical City, Ministry of National Guard ‑ Health Affairs, Riyadh, SAU

**Keywords:** rhabdomyoblastic differentiation, pelvic sarcoma, post-radiation sarcoma, radiation-induced sarcoma, rhabdomyosarcoma

## Abstract

Radiation-induced sarcomas (RIS) are a rare long-term complication of radiation therapy, with a reported incidence of 2.5-5.5%. They usually develop several years following exposure to radiotherapy. The most common reported subtypes are undifferentiated pleomorphic sarcoma, angiosarcoma, and leiomyosarcoma. Breast cancer is the most common primary malignancy preceding RIS, followed by uterine cervical carcinoma. Only a few cases of RIS with rhabdomyoblastic differentiation have been reported in the literature, usually following the treatment of retinoblastoma. Herein, we report a rare case of RIS with rhabdomyoblastic differentiation in the pelvic region developing 12 years after cervical cancer radiation therapy.

## Introduction

Radiation-induced sarcomas (RIS) are a rare but well-known complication of radiation therapy. Most cases have been described following the treatment of breast and uterine cervical cancers [1]. The most common subtypes are undifferentiated pleomorphic sarcoma, angiosarcoma, and leiomyosarcoma [2], with only a few reported cases of RIS with rhabdomyoblastic differentiation. Herein, we report a case of a 48-year-old female with RIS with rhabdomyoblastic differentiation of the uterine cervix after 12 years of latency.

## Case presentation

A 48-year-old female presented to the emergency department with hip pain after withstanding a fall. On further investigation, the patient was found to have a 12-year history of moderately differentiated squamous cell carcinoma of the uterine cervix. She was treated with chemo-radiotherapy with a complete response and no residual disease; however, she was lost to follow-up after that.

**Figure 1:**
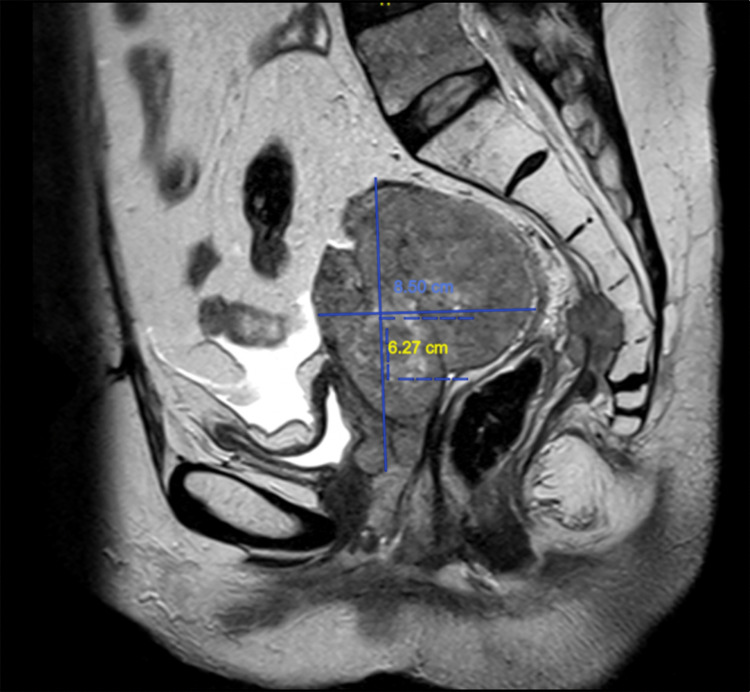
Pelvic MRI

On examination, the vulva and vagina were involved by multiple, irregular masses of varying sizes that bled on touch. The cervix was not visible. Pelvic MRI showed an aggressive mass involving the uterus, vagina, perineum, distal rectum, proximal anal canal, and posterior wall of the urinary bladder. The mass extended through the right greater sciatic foramen with involvement of the gluteal muscles (Figure 1).

**Figure 2:**
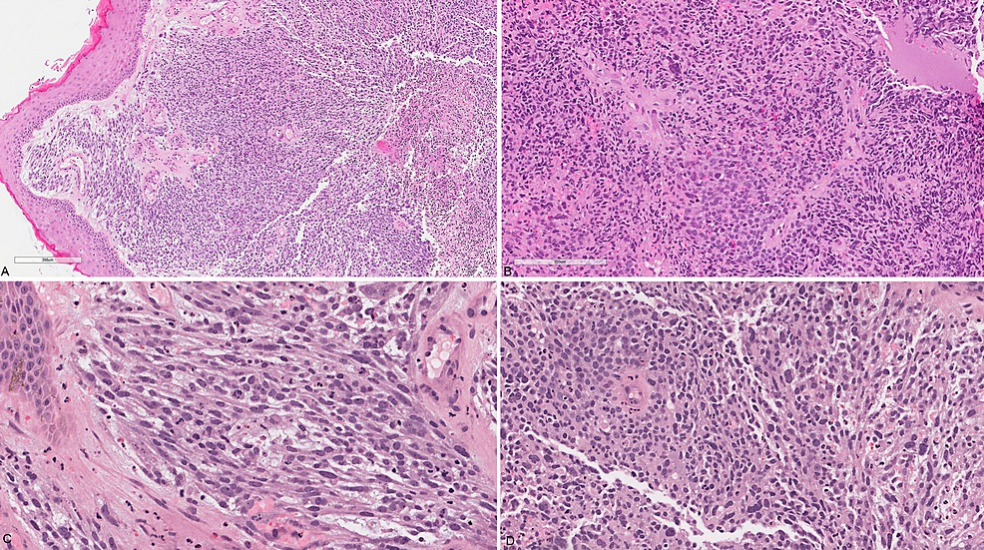
Valvular biopsy (A) Low power view showing unremarkable squamous epithelium overlying sheets of neoplastic cells; (B) diffuse sheets of pleomorphic plump to spindle neoplastic cells; (C) and (D) high power view of the spindle cell areas showing brisk mitosis and focal possible rhabdomyoblastic differentiation.

**Figure 3:**
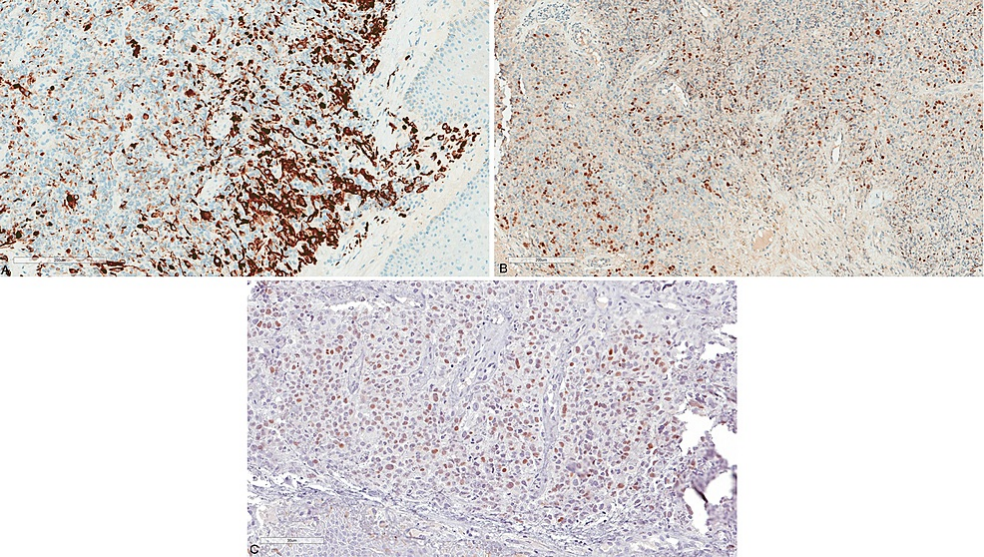
Immunohistochemistry of the neoplastic cells (A) Cytoplasmic staining for desmin; (B) patchy strong nuclear staining for myogenin; (C) MyoD1.

A vulvar biopsy was taken, and it showed fragments of unremarkable squamous epithelium overlying sheets of malignant high-grade pleomorphic epithelioid and spindle cells (Figure 2A and 2B). The cells have a high nuclear to cytoplasmic ratio, vesicular nuclei, and inconspicuous nucleoli. Some of the cells had a deeply eosinophilic cytoplasm raising the possibility of rhabdomyoblastic differentiation (Figure 2C and 2D). Several foci of necrosis are seen, as well as brisk mitotic activity. By immunohistochemistry, the neoplastic cells were positive for desmin (DE-R-11; Figure 3A), myogenin (F5D; Figure 3B), and myoD1 (EP212; Figure 3C); and negative for Pan-CK (AE1/AE3), CK5/6 (D5/16B4), EMA, p63 (4A4), p16 (E6H4), LCA (2B11+PD7/26), S100 (polyclonal), caldesmon (h-CD), and SMA (1A4). Based on the clinical history, tumor morphology, and immunoprofile, a diagnosis of post-radiation sarcoma with rhabdomyoblastic differentiation was rendered.

The patient received several cycles of chemotherapy and local radiation therapy with no significant response. Her options were discussed in the tumor board, and the tumor was deemed unresectable. She then was referred for palliative radiation therapy and pain control, where she succumbed to her disease shortly thereafter.

## Discussion

Radiation therapy plays a critical and integral role in the treatment of many cancers. The advances in cancer management have led to improved survival and increased life expectancy. As a result, radiation-induced secondary malignancies are an emerging concern that is expected to rise in the upcoming years [3,4].

Radiation-induced sarcomas are a well-known, albeit rare complication, with an incidence of approximately 2.5-5.5% in most reported series [5]. One of the hallmarks of radiation-induced malignancies is latency. Cahan and Woodard described the development of RIS in 11 patients five to more than 22 years following exposure to radiotherapy [6]. It is believed that latency is inversely proportional to radiation dose; however, a clear-cut relationship is yet to be established [1].

A large study from Memorial Sloan Kettering Cancer Center reviewed 130 cases of primary RIS defined using a modification of the criteria proposed by Cahan and Woodard. These criteria included: (1) history of radiation exposure at least six months before the development of sarcoma, (2) occurrence of sarcoma within the radiation field, and (3) pathologic confirmation of a sarcoma that was histologically different from primary cancer. In this series, Gladdy et al. found that the most common histologic type of RIS is undifferentiated pleomorphic sarcoma (previously known as malignant fibrous histiocytoma), followed by angiosarcoma, leiomyosarcoma, and fibrosarcoma [2].

Few cases of RIS with rhabdomyoblastic differentiation have been described in the literature. Dang et al. reviewed 43 cases of rhabdomyosarcomas arising in a previously irradiated field, examining their characteristics and outcomes. The most common initial tumor was retinoblastoma (in 42% of patients) followed by breast cancer and only three cases of cervical cancer. The median latency period was eight years, with a range from 1.3 to 30 years. Most RIS rhabdomyosarcomas were treated surgically, other treatment modalities included chemotherapy, radiotherapy, or a combination of systemic and local treatment. In their review, the three-year overall survival rate was 42% [7].

Wide surgical resection is the treatment of choice for RIS. Unfortunately, since these patients are usually diagnosed with advanced disease, approximately 50% of cases are inoperable [3]. Compared to primary soft tissue sarcomas, RIS has an aggressive behavior with a worse overall prognosis [8]. This is likely due to their central location, incomplete surgical resection, and presence of metastasis [5].

## Conclusions

In conclusion, RIS is a rare but rising concern that must be kept in mind whenever faced with a malignant neoplasm in a previously irradiated field. A high index of suspicion is needed to correctly diagnose these cases, aided by the appropriate use of immunohistochemical stains. Although the most common subtypes are undifferentiated pleomorphic sarcomas and angiosarcomas, other rare subtypes should also be included in the differential diagnosis. The importance of RIS lies in the fact that their prognosis is worse than primary soft tissue sarcomas.

## References

[1] Laskin WB, Silverman TA, Enzinger FM (1988). Postradiation soft tissue sarcomas. An analysis of 53 cases. Cancer.

[2] Gladdy RA, Qin LX, Moraco N (2010). Do radiation-associated soft tissue sarcomas have the same prognosis as sporadic soft tissue sarcomas?. J Clin Oncol.

[3] Karunanithi G, Sethi P, Reddy K, Vivekanandam S (2008). Radiation induced sarcoma of pelvis. Internet J Oncol.

[4] Kim KS, Chang JH, Choi N (2016). Radiation-induced sarcoma: a 15-year experience in a single large tertiary referral center. Cancer Res Treat.

[5] Bjerkehagen B, Småstuen MC, Hall KS, Skjeldal S, Smeland S, Fosså SD (2012). Why do patients with radiation-induced sarcomas have a poor sarcoma-related survival?. Br J Cancer.

[6] Cahan WG, Woodard HQ, Higinbotham NL, Stewart FW, Coley BL (1998). Sarcoma arising in irradiated bone: report of eleven cases. 1948. Cancer.

[7] Dang ND, Teh BS, Paulino AC (2013). Rhabdomyosarcoma arising in a previously irradiated field: an analysis of 43 patients. Int J Radiat Oncol Biol Phys.

[8] Bloechle C, Peiper M, Schwarz R, Schroeder S, Zornig C (1995). Post-irradiation soft tissue sarcoma. Eur J Cancer.

